# Partitioning of the nervous system following exoskeleton and epidural stimulation in spinal cord injury

**DOI:** 10.1038/s41598-026-52650-0

**Published:** 2026-05-12

**Authors:** Ashraf S. Gorgey, Momal Ahmad Wasim, Jakob N. Deitrich, Muhammad Uzair Rehman, Carrie Peterson, Robert Trainer

**Affiliations:** 1 Spinal Cord Injury and Disorders, Richmond VA Medical Center, 1201 Broad Rock Blvd, Richmond, VA 23249 USA; 2 Physical Medicine and Rehabilitation, School of Medicine, Richmond, VA USA; 3 Department of Biomedical Engineering, Virginia Commonwealth University, Richmond, VA 23284 USA; 4 Physical Medicine and Rehabilitation, Richmond VA Medical Center, Richmond, VA 23249 USA

**Keywords:** Percutaneous SCES, Central nervous system, Peripheral nervous system, Exoskeletal-assisted walking EMG, 10-m walk test, Velocity dependent spasticity, SCI, Medical research, Neurology

## Abstract

The effects of percutaneous spinal cord epidural stimulation (SCES) on motor control have yet to be elucidated in persons with spinal cord injury (SCI). We examined the effects of percutaneous SCES on the central and peripheral nervous systems after SCI. We determined the effect of SCES combined with exoskeletal assisted walking (EAW) and resistance training (RT) on motor performance as assessed by the 10 m-walking test, electromyography outcomes and spasticity measurement. Four chronic (6–24 years post-injury) motor complete (C8-T11 AIS A and B) persons with SCI underwent permanent implantation with percutaneous SCES. Participants were either assigned to 12 months of EAW + SCES or EAW without SCES, 3 × weekly for the first 6 months. This was followed by an additional 6 months of EAW + SCES + RT (*n* = 3) or EAW + delayed SCES + no RT (*n* = 1) (both groups: 3–5 × weekly). Measurements were conducted at baseline (BL), post-intervention 1 (P1, 6 months following BL) and post-intervention 2 (P2, ~ 12 months following BL). Central nervous system performance was evaluated by measuring volitional knee isometric torques with SCES ON compared to SCES OFF. H-max/M-max ratio was measured for peripheral nervous system performance. For central adaptations, two participants (0882 and 0884) intentionally (SCES ON + active movement) generated knee extensor isometric torques at either 75% sub-motor threshold (0882; 1.87–6.6 × greater than SCES ON) or 100% motor threshold [0882 (1.6 × greater than SCES ON) and 0884 (3.7–16 × greater than SCES ON)]. A third participant initiated torques in the flexion direction and the fourth participant failed to initiate isometric torque. For peripheral adaptations, individual data indicated improvement in H-reflex and M-waves following P1 (0881 and 0882) and P2 (0881 and 0884). Surface neuromuscular electrical stimulation induced greater (24–42%) isometric knee extensor torques at P2 compared to BL in both groups. Overall, 10-m walking test resulted in 30% longer duration and 29% slower speed with reduced EAW assistance (50–70%) during EAW + SCES ON (duration: 79 s and speed: 0.13 m/s) compared to 100% EAW + SCES OFF (duration: 60.5 s and speed: 0.18 m/s) during P1 measurements. In P2, similar pattern was observed and characterized by longer duration (22%) and slower walking speed (24%). Spasticity decreased (0.5–13%) at different angular velocities following P1 but not after P2. EAW + SCES ON improved intention to generate isometric torques accompanied with improvement in peripheral neuromuscular kinetics. This was mirrored by increasing EMG harmony with training without enhancing EAW 10-m performance. The effects of EAW + SCES on spasticity may warrant additional investigation.

**Supplementary Information:**

The online version contains supplementary material available at 10.1038/s41598-026-52650-0.

## Introduction

Percutaneous spinal cord epidural stimulation (SCES) has been previously employed to manage pain syndromes and spasticity^1–3^. Applications of percutaneous SCES have gained considerable attention and have effectively translated from several clinical trials to become a real tool in a clinical setting^1–4^. Unlike pain management, applications of percutaneous SCES for motor recovery following several neurological disorders are still not universally accepted^5–7^. This is despite paddle implantation demonstrating that SCES facilitates access to the lumbar epidural space to induce activation of the corresponding neural spinal cord circuitries^8–12^. Research findings indicated that SCES may provide access to spinal cord locomotor centers via integrating proprioceptive sensory input into motor outcomes^8,9^. Target stimulation of specific motor unit pools resulted in restoration of motor control to achieve standing and walking in persons with spinal cord injury (SCI)^10–12^. Two major SCES techniques are currently targeting restoration of motor recovery. The first relies on enabling the lumbosacral locomotor centers in conjunction with task specific training to restore motor control after SCI^10–12^. The second is targeting the lumbosacral spinal cord circuitries to induce spatiotemporal stimulation in real time to initiate stepping during the gait cycle^13–15^. Today, limited knowledge is available on how percutaneous SCES may restore motor control after SCI. Additionally, the findings from paddle-based studies may not be generalized to percutaneous SCES.

Our group has previously shown that percutaneous SCES enables motor control in two different persons with complete and motor complete SCI^7,16^. One trial demonstrated that a T11 person with an AIS B SCI could independently stand and walk using a standard roller walker^7^. Another case report demonstrated interleaved configurations of the percutaneous SCES decreased adductor muscle tone and resulted in improvement of overground stepping in a person with complete T3 AIS A SCI^16^. Another case report indicated that percutaneous SCES-induced rhythmic configurations elicited reduction in spasticity in a person with C6 AIS A SCI^17^. Together, these reports demonstrated that restoration of motor performance is potentially an integration of the central nervous system (CNS) and/or the peripheral nervous system (PNS). In the context of the current work, CNS refers to the motor cortex, sub- motor cortex and spinal cord, whereas PNS reflects spinal cord motor units, peripheral nerves and skeletal muscles. Several mechanistic reports indicated that SCES may integrate the descending central supraspinal control signals with the peripheral afferent kinesthetic signals to enhance motor control^18–20^. These findings collectively suggest that percutaneous SCES may elicit both CNS and PNS adaptations in persons with SCI.

The exact mechanism (s) by which percutaneous SCES restores motor control has yet to be deciphered. It is still unclear whether CNS and/or PNS adaptations may be necessary to restore motor recovery similar to standing or walking. Previous reviews indicated that motor recovery in humans with SCI is plausible without descending supraspinal control^18–20^. This may suggest that improvement in PNS afferent input may induce motor recovery without reliance on supraspinal control. However, others indicated that descending supraspinal control is essential for motor recovery^15^. This is supported by previous murine model findings that indicated the regeneration of axons across the level of complete SCI enhanced supraspinal control to restore waking^15^. Therefore, it is plausible to assume that maximizing motor performance following percutaneous SCES relies on descending supraspinal control integrated with optimized afferent peripheral inputs.

To address this hypothesis, knee extensor isometric torque was used as a proxy index to reflect supraspinal descending CNS intention with SCES ON. Additionally, H-max/M-max ratio and surface neuromuscular electrical stimulation (NMES) were used to examine the afferent and motor unit connectivity as well as the contractile machinery properties of the knee extensor muscle. Finally, the translation of CNS and PNS motor performance was determined via 10-m exoskeleton assisted walking (EAW) performance with and without SCES, changes in EMG following training and changes in the spasticity-magnitude at different angular velocities. Therefore, the purpose of the current work was to examine the interplay between supraspinal central input and PNS following implantation of percutaneous SCES in persons with chronic SCI. We hypothesized that participants who demonstrate changes in the CNS and/or PNS following SCES implantation may experience improvement in motor performance after SCI.

## Methods

### Human subjects

A detailed study timeline was recently published demonstrating all aspects of the trial as well as the inclusion–exclusion criteria^21^. Four men with clinically motor complete traumatic SCI (Table 1) participated in a randomized pilot clinical trial that was approved by the Richmond Veterans Affairs Medical Center^21^. All methods were performed per the relevant guidelines and regulations, and the trial was registered at clinicaltrials.gov, with registration I.D. # NCT04782947. Data collection started in February 2021, anticipating continuing enrollment until the end of 2026. All participants provided informed written and verbal consent to all study procedures that were approved by a local ethics committee. After consenting, all participants underwent detailed physical and medical screenings by a certified SCI physician including International Standards for Neurological Classification of Spinal Cord Injury (ISNCSCI) as well as modified Ashworth scale assessment (Table 2).

**Table 1:** Physical and clinical characteristic of individuals with SCI who were randomized into the REST-SCI trial.

Participant ID#	Sex	Age (yrs.)	Weight (kg)	Height (cm)	BMI	TSI (years)	NLI	AIS	Classification	Randomization	Completion	Measurements	Implantation
0881	M	25	48.6	174.3	16.0	6	C8	A	Tetraplegia	EAW + ES + RT	Completed	BL, P1, P2	Prior to BL
0882	M	36	99.4	182.2	29.9	9	T11	B	Paraplegia	EAW + ES + RT	Withdraw following 10 months	BL, P1	Prior to BL
0883	M	38	99.0	180.5	30.4	12	T6	A	Paraplegia	EAW + delayed ES + noRT	Completed	BL, P1*, P2	6 months after BL
0884	M	54	93.4	182.8	28.0	24	T4	A	Paraplegia	EAW + ES + RT	Completed	BL, P1, P2	Following BL

Yrs., Years; kg, Kilograms; cm; centimeters; kg/m^2^, Kilograms per meter squared; BMI, Body mass index; TSI, Time since injury; AIS, American Spinal Injury Association Impairment Scale; M, Male; NLI, Neurologic level of injury.

*Biodex dynamometer was not functioning before P1 in 0883.

**Table 2:** Modified Ashworth Scores (MAS) at the time of admission in the REST-trial.

Participant ID#	Hip flexors-R	Hip flexors-L	Knee extensors-R	Knee extensors-L	Knee flexors-R	Knee flexors -L	Calf muscles-R	Calf muscles-L	Ant-spastic medications
0881	1 +	1 +	1 +	1 +	3	3	3	3	None
0882	–	–	2	2	2	2	2	2	None
0883	1	1	–	–	2	2	1	1	None
0884	0	0	1	1	0	0	2	2	None

Grade 0: No increase in muscle tone; Grade 1: Slight increase in muscle tone with slight catch and release or minimal resistance at end of stretch; Grade 1 + : Slight increase in muscle tone with minimal resistance after catch that lasts throughout remainder of range of motion; Grade 2: Moderate increase in muscle tone but affected part still easily moved; Grade 3: Considerable increase in muscle tone with difficulty in passive range of motion; R:right limb; L: left limb. Dash (–): values were not recorded.

### Inclusion–exclusion criteria

Briefly, all participants were between 18 and 60 years old, male or female, with traumatic motor complete SCI (AIS A and B) and level of injury of below C5 as determined by International Standards for Neurological Classification of SCI (ISNCSCI) exam. Participants must be at least 2 years post-injury to be considered for the trial. A written clearance by the medical provider was provided to ensure safety prior to enrollment in the program.

Participants with any of the following pre-existing medical conditions were excluded from the current trial: (1) diagnosis of neurological injury other than SCI, including cauda equina or distal conus injuries resulting in limb or sacral are flexia; (2) unhealed fracture in either lower or upper extremities; (3) severe scoliosis, hip knee range of motion or flexion knee contractures preventing positioning in an exoskeleton or plantarflexion contracture greater than 20 degrees; 4) untreated or uncontrolled hypertension defined as high resting blood pressure greater than 140/90 mmHg and severe orthostatic hypotension (drop greater than 20 mmHg compared to resting supine blood pressure) or incapable to maintain a sitting or EAW standing posture; 5) other medical conditions including cardiovascular disease, uncontrolled type II DM, and those on insulin, or symptomatic urinary tract infection; 6) taking anti-coagulants or anti-platelet agents, including aspirin if unable to be off this medication for medical reasons; 7) implanted pacemakers and/or implanted defibrillator devices; 8) DXA T-scores or BMD less than − 2.5, − 3.5 SD and 0.6 g/cm^2^, respectively, for total body, dual hips and knees. 9) participants with severe spasticity or limited ROM as measured by modified Ashworth scale; 10) pressure ulcer of the trunk, pelvic area, or lower extremities of grade 3 or more; 11) psychopathology documentation in the medical record or history that may conflict with study objectives; 12) Allergic reaction to antibiotics minocycline and rifampin; 13) pregnant women and women who may become pregnant during the course of the study; 14) any condition that, in the judgment of the principal investigator or medical provider, precludes safe participation in the study and/or increases the risk of infection.

### Timeline of the study

**Fig. 1:**
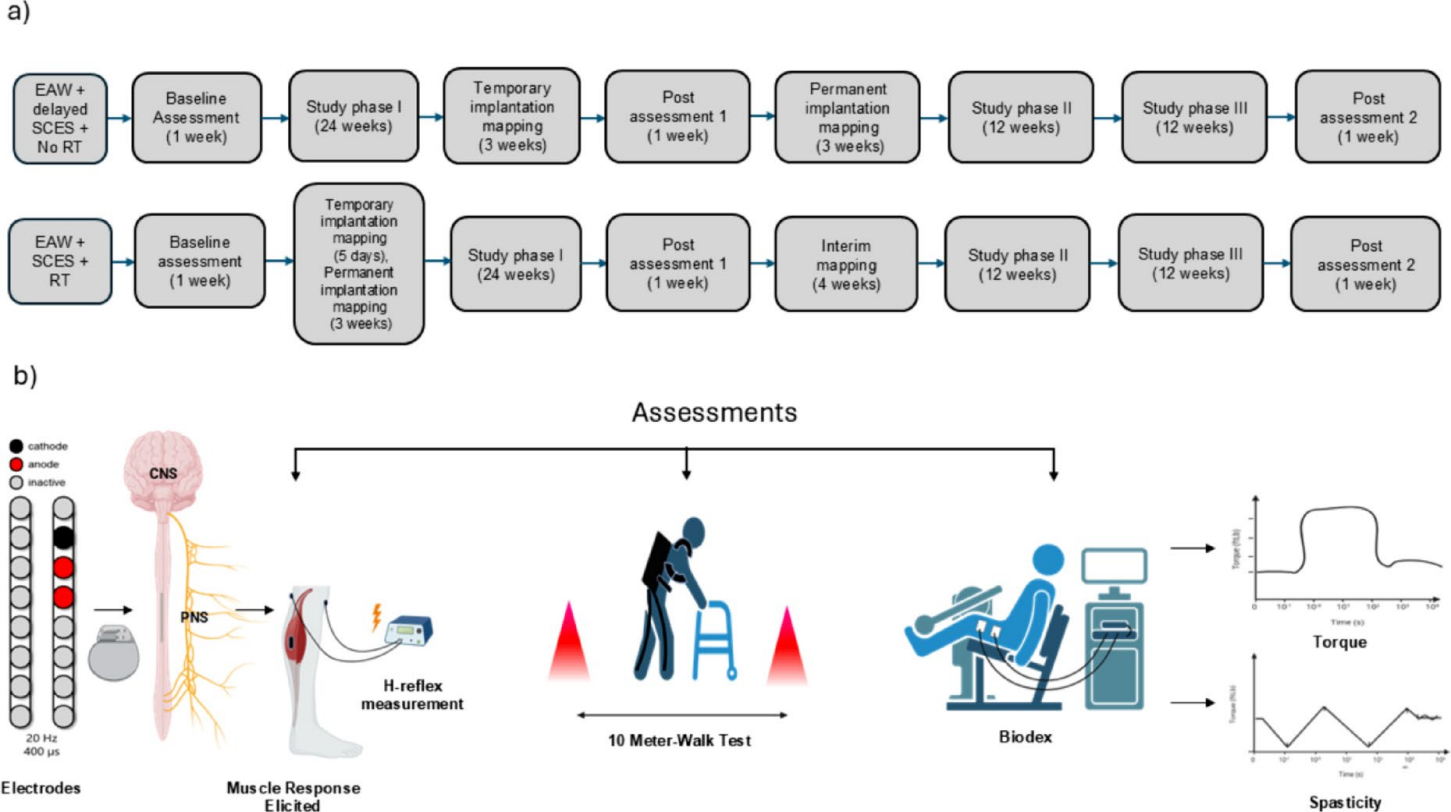
A schematic diagram represents the timeline and the design of the current trial. After recruitment, participants underwent implantation of percutaneous SCES. This was followed by performing measurements at three different time points (BL, P1 and P2) separated by approximately 6 months apart. (**a**) Highlights the timeline of both groups based on the randomization into either EAW + SCES + RT or EAW + delayed SCES + No RT. (**b**) The figure highlights procedures of testing both CNS and PNS adaptations. CNS adaptation was tested by the volitional intention of the participant to voluntarily initiate isometric torque with SCES OFF and SCES ON. PNS system adaptations were measured via eliciting (**a**) H-reflex/M-wave of the soleus muscle and (**b**) with surface NMES (not shown) to elicit electrically induced isometric torques at different frequencies (20, 40 and 80 Hz). Finally, the outcomes of motor performance were examined via (**a**) EAW 10-m walk test, (**b**) EMG activities of different muscle groups and (**c**) spasticity-resistance responses to different angular velocities for the knee extensor muscle group.

Seven participants have been enrolled in the current trial (4 male participants completed the trial, 2 participants withdrew immediately after signing consent forms, 1 participant was considered screen failure). Briefly, age range was 25–54 years old, with body weight of 48.6–99.4 kg and level of SCI of C8-T11 AIS A and B. The participants were enrolled in the study for approximately 12 months with the purpose of examining the effects of implanted percutaneous SCES in conjunction with EAW and task specific training including stand training and resistance training (RT) for restoration of motor control after SCI. The timeline of the entire study was previously described in detail^21^ and is listed in Fig. 1a. Following implantation, participants underwent detailed spinal cord mapping at different phases of the study^7,22^. The purpose of spinal mapping was to determine the exact SCES configurations and stimulation parameters^21–24^.

Three participants were randomized into 6 months of EAW + SCES followed by an additional 6 months of adding RT to EAW + SCES (EAW + SCES + RT; participants # 0881, 0082 and 0884). Surface neuromuscular electrical stimulation (NMES) was used for 12 weeks to execute the RT protocol similar to previous work^25^. RT was performed with SCES OFF followed by 12 weeks of closed-chain RT in the form of sit-to-stand activity with SCES ON^21^. The remaining participant was randomized into 6 months of EAW only followed by 6 months of implanting percutaneous SCES without adding RT (EAW + delayed SCES + noRT; participant # 0883). Briefly, the rationale of this design was based on enhancing motor outcomes via the implantation of percutaneous SCES and later the addition of NMES-RT to evoke muscle hypertrophy. Evoking muscle hypertrophy is likely to maximize the afferent loops to locomotor spinal centers, which is necessary to further enhance the outcomes of SCES on PNS and motor recovery^21^.

All participants underwent 60 min of EAW training 3 × weekly across the entire study. Following implantation, additional 60 min of stand-specific training were added to the EAW for all participants^21^. The stand-specific training primarily focused on sit-to stand activity and maintaining standing balance using a walker, parallel bars or standing frame. Other exercise activities have been introduced based on the needs of each participant and the level of injury. Measurements were completed at baseline (BL), post-intervention 1 (6 months from BL) and post-intervention 2 (12 months from BL).

## Implantation of percutaneous SCES

Each participant underwent two phases of implantation starting with the temporary trial phase and followed by permanent phase (3–4 weeks following temporary implantation) using the SCES system (Intellis Epidural Stimulator, Medtronic, Minneapolis, USA)^7,17,21^. Step-by-step procedure of both phases of implantation was previously published in detail^7,21^.

Briefly, temporary implantation was conducted to determine possible unforeseen problems that could lead to withdrawal from the trial or disqualify the participant from undergoing the permanent procedure. The epidural space was accessed through 14-gauge epidural needles using the loss of resistance technique with X-ray assistance. The leads were guided to both sides of the midline on live fluoroscopy to confirm posterior epidural placement. Following initial testing via fluoroscopy, the lead position was optimized after confirmation of proper motor stimulation. The needles in the epidural space were removed^7,21^. Electrodes were secured to the skin with tape and glue to decrease the possibility of lead migration. EMG spinal mapping was performed on the same day or the following 3 consecutive days^7,21^.

For permanent implantation, two 8-electrode arrays of Vectris leads were implanted 3–4 weeks after temporary implantation^7,21^. An anesthesiologist performed IV sedation, the epidural space was then accessed through 14-gauge epidural needles using the loss of resistance technique with X-ray guidance. After a vertical incision was made in the lateral low back between the 12th rib and iliac crest, a pocket was dug out between skin and muscle for the battery with bovie and scissors and the pulse generator were then placed in the pocket. The leads were secured with non-absorbable 0 monocryl sutures to interspinal ligament and/or lumbodorsal fascia.

The leads were guided under the skin to the pocket of tissue, where they were then connected to a Medtronic Intellis battery. Impedances were checked after hemostasis was complete and irrigation applied. Derma-bond, occlusive dressing, and tape were placed over the wound. An abdominal binder was made available to the participant to increase comfort. Bandages were removed at the 7–10-day mark after implantation when recovery was determined to be complete and incision site was examined for the first month. Participants were instructed to avoid strenuous physical activities without immobilization for the first 3–4 weeks^7,21^.

### Spinal mapping

Spinal mapping was conducted daily following both temporary (3–5 days) and permanent (2–3 weeks) implantation after 3–4 weeks of non-strenuous physical activity^22,23^. In this process, various electrode configurations consisting of different anode/cathode configurations, pulse widths, and stimulation frequencies were tested to achieve a multitude of functions without unwanted movements. Optimal electrode configurations were determined, including stimulation frequencies (2 Hz) and pulse duration (250–1000 µs). The minimum amount of current (1–12 mA) was applied to evoke desirable motor activity in the target muscles. Initially, spinal mapping was targeted towards achieving standing in the first 6 months. Following P1, spinal mapping was targeted toward achieving both standing and stepping^21–23^.

We initially specified the configurations that target individual muscles in the supine lying position. These configurations were tested to ensure specific joint movements in a supine lying position. We then tested these configurations in a standing position using either a standard walker or a standing frame. The configurations were then modified based on personal needs to attain functional goals. We then adopted standardized procedures that were previously described in detail^16,21–23^ to primarily focus on achieving standing posture in the first 6 months. This is followed by an interim mapping to configure the lumbosacral rhythmicity to enhance stepping in the remaining 6 months of the trial^21–23^.

Lead migration is a potential consequence following percutaneous SCES implantation. Migration is likely to influence target segmental stimulation and may attenuate functional gains. We used either fluoroscopy or spine dual-energy x-ray absorptiometry to measure medio-lateral or caudal-cephalic migration over time^7,23^. Suppl Table 1 reflects the SCEC configurations and stimulation parameters for different outcome variables throughout the entire study.

### EMG data recording and analysis

In the supine position, after shaving and cleaning the skin, ten EMG sensors were attached to the following muscles: left and right vastus medialis (VM), left and right rectus femoris (RF), left and right hamstrings (HS), left and right tibialis anterior (TA), and left and right medial gastrocnemius (MG). The participant was asked to move the entire leg into flexion and extension, dorsiflexion of the ankle joint, and wiggling the big toe. At both temporary and permanent mapping, various electrode configurations with different stimulation parameters were tested to achieve optimal activation of the paralyzed leg muscles^21–23^. A video supplementation was recently published highlighting the entire procedures of initiating movement with SCES at different joints^21^.

Surface EMG activity was recorded for the key muscles including RF, VM, HS, TA and MG. All EMG signals were collected at a 2000 Hz sampling rate using LabChart 8.1.21 (Windows, A.D. Instruments, Sydney, Australia). Root Mean Square (RMS) was used to quantify the amplitude of muscle activation over a 5-s interval in response to each stimulation amplitude and for each stimulation configuration^21–23^. Resting EMG activity was quantified along a 5-s interval without any stimulation for each channel and used to adjust baseline drift during temporary and permanent mapping. All temporary and permanent mapping motor evoked responses were normalized to the maximum value for each individual muscle across all configurations. Recruitment curves were developed to analyze the progression in muscle evoked potential amplitude in response to increasing stimulation amplitude^22,23^.

## Interventions

### Exoskeleton assisted walking (EAW)

A member of the research team assisted in fitting the participant into the EAW unit (EksoNR, Ekso Bionics, CA, USA) starting with the shoes-support and then going up toward the trunk in a sitting position^7,24^. EAW was scheduled for 3 days/week for 12 months and the software was adjusted and progressed based on the needs of each participant to facilitate walking. All supportive straps were well adjusted but not overly tight to avoid development of unnecessary episodes of autonomic dysreflexia^24^. Initially, training started in the first-step mode with a roller walker until participants mastered to shift body weight anterolaterally to achieve stepping.

Participants were then progressed to EAW pro-step + mode starting with a standard roller walker and then transitioned to Canadian Crutches in approximately 4 weeks. The EAW support-assistance ranged from 0–100%, where 100% indicated that the unit provided the maximum support and assistance for ambulation. During the first 6 months, the EAW assistance was set to adaptive mode for 10–15 min for warming up purpose followed by fixed assistance for the entire session. For the remaining 6 months (months 7–12), the EAW was completely switched to the adaptive mode to provide the necessary freedom to modulate the level of assistance based on performance^24^.

For fixed assistance, participants were encouraged to turn on their SCES and the EAW- assistance was gradually decreased in 5% increments until attaining the lowest assistance level during the first 6 months^24^. The decision to drop the EAW assistance was based on the ability of the participant to complete 80% (i.e. arbitrary threshold) of the steps during a10-m distance without cueing from exoskeleton. After dropping the assistance, the participant was provided with only 2.4 s to actively step, before the exoskeleton passively moved the leg. A research assistant using two different digital counters to count the number of active steps (i.e. participant) compared via passive steps (i.e. exoskeleton) using exoskeleton audible beeps^24^. The audible beeps only triggered when the participant failed to initiate stepping within 2.4 s. We proceeded with the decision to drop the EAW assistance if the number of active steps equal or exceeded 80% of the total number of steps during the 10-m walking test^24^.

The EAW unit was equipped with two buzzers that helped to cue the participants to accurately complete weight shifting prior to stepping. A trained research assistant provided guidance and support from the back during EAW training session. Participants were encouraged to complete 60–90 min training session including fitting time, resting, and walking time. Resting and EAW vital signs (heart rate, blood pressure and %SO_2_) were monitored every 5 min to ensure safety^21,24^.

### Stand training

Following permanent implantation, each participant was also involved in 60 min stand specific training period for 3 × weekly including sit-to-stand activity, back extension exercise for trunk control while using a standard walker or a standing frame. Back extension exercise was only performed when trunk control is limited or deficient in our participants. The standing frame encouraged trunk control via encouraging participants to control hip extension with locking their knees. The training paradigm was then advanced to use the parallel bars and then standard walker. Two research assistants worked with each participant to enable trunk control and to secure independent standing with SCES ON as previously described^7,21,22^. The stand-specific training protocol was tailored to ensure functional gains and based on the progress of each participant across the trial^7^.

### *Resistance training (EAW* + *ES* + *RT group)*

Following P1, resistance training (RT) was conducted twice weekly only for the EAW + ES + RT group^21,25^. RT was performed in an open-chain format for the first 12 weeks using surface NMES^25^ and followed by another 12 weeks of closed-chain format via using the implanted SCES. Two 8 × 10 cm^2^ (Uni-Patch, Wabasha, MI, USA) adhesive carbon electrodes were placed on the skin over the knee extensors. Surface NMES was unilaterally applied to the knee extensor muscles via surface electrodes to induce concentric-eccentric actions starting with the right and followed by the left leg after resting period of 2–3 min between legs^25^. The NMES-RT protocol was conducted via using commercially available ankle weights that were progressed with an increment of 2 lbs. on a weekly basis^25^. The NMES current was gradually ramped in 5-s intervals until knee extension was achieved against gravity followed by gradually decreasing the current to induce lengthening actions until full relaxation (i.e. return to the resting position). Each RT session consisted of 4 sets of 10 repetitions of NMES-induced knee extensions for 45–60 min. A 5 s/5 s work/rest ratio was used with a 3-min rest between sets, 30 Hz, 450 µs pulses and a current sufficient to evoke full knee extension^21,25^. Each participant performed an additional 12 weeks of closed-chain RT (i.e. gradual sit-to-stand exercise using a standard walker) via using their implanted SCES after seated on an adjustable mat and turning on SCES using a standard walker with two research assistants under full supervision.

## Measurements

All 4 participants underwent the listed measurement procedures to evaluate the effects of EAW + SCES on CNS, PNS and motor perfomance (Fig. 1b).

### Motor adaptations

#### 10-m EAW speed, EAW unassisted steps and EMG pattern

Walking time and speed using the 10-m walk test (10-MWT) was determined using either a walker or crutches^7,21,24^. Electromyography (EMG) activity was recorded from hip, knee and leg muscles during locomotion unilaterally by bipolar surface electrodes with fixed inter-electrode distance (Delsys Inc. Massachusetts, USA. A calibrated electric goniometer with three-foot sensors were used to dissect the gait cycle into stance and swing phases^24^. The procedure was done prior to implantation and every 6 months after implantation. During the 10-MWT, participants performed the walk between two cones set 10 m apart (Fig. 1), this was repeated with SCES ON and without SCES (SCES OFF).

The sequence started with SCES OFF and then with SCES ON, allowing each participant to serve as his own control^7,21^. The SCES artifacts were minimized from EMG data with a bandpass filter (10–990 Hz) using custom Lab Chart 8.1 (ADInstruments, Sydney, Australia). For baseline (BL) and post-intervention (P1 and P2) data, the filtered and continuous data were then visually segmented into individual strides by using the vertical component of an accelerometer embedded within the EMG sensors^7^. The data were segmented using the anteroposterior gyroscopic signal embedded within the EMG sensor of the gastrocnemius muscles. Each stride was further segmented into stance and swing phases of the gait cycle.

EMG RMS envelopes were calculated using a moving window of 150 samples for 10 strides^23^. Peak filtered EMG values across the entire stride, stance phase, and swing phase were identified and averaged over the total number of strides completed during different assistance levels. All EMG activities were normalized to EMG activities of EAW-100% assistance for different muscle groups and at different timepoints (BL, P1 and P2 measurements). A criterion method of greater than 1.0 was used to determine whether EMG activity increased or decreased following training. Using a counter, a researcher counted the number of EAW unassisted steps (i.e. active steps without audible cueing) during the 10 MWTs as well as the walking speed in a similar fashion as described above^24^.

### Neurophysiologic assessments

One week prior to intervention (BL), and during P1 and P2**,** segmental sensorimotor reflexes were studied by recording H-reflexes only with SCES OFF. The H-reflex: M-wave ratio was used to determine the effects of the training on the PNS^26,27^. The soleus H-reflex was recorded (20 Hz–20 kHz bandpass) with a surface EMG electrode on the motor point of the soleus muscle with the subject in a standing position using the exoskeletal standing mode and a standard walker to support upper body. Exoskeletal standing mode was set at maximum support and consistently repeated at BL, P1 and P2 measurements to ensure controlling for lower limb loading. All participants equally tolerated H-reflex measurements in a standing position.

Briefly, electrical stimulation (1-ms duration pulses) was applied using a constant current stimulator (DS7AH, Digitimer Ltd.) at 0.5 Hz to the tibial nerve at the popliteal fossa by a monopolar electrode. Stimuli were manually delivered after gradually increasing the amplitude of the current to obtain H-reflex of maximal amplitude (H-max). Each electrical stimulus was manually delivered three times with a resting interval of 5 s between two incremental stimuli to attenuate the likelihood of developing frequency dependent depression of H-reflex^28^. Each point represents the mean peak-to-peak amplitude of the three reflexes that elicited at different current intensity (1–40 mA). The amplitude and latency of H-max were measured. The intensity at which the M-wave of maximal amplitude (M-max threshold) was recorded^26,27^. The ratio of H-max to M-max was calculated at BL, PI and P2 to determine the effects of the intervention on the PNS.

### Spasticity and knee extensor torque

Knee extensor spasticity and isometric torque for both groups were evaluated using a Biodex isokinetic dynamometer (Shirely, NY) consistently across the 4 participants^7,17,21^. Participants were seated with both the trunk-thigh angle and the knee-thigh angle at 90°^17^. After transferring using a ceiling lift, each participant was securely strapped to the test chair by crossover shoulder harnesses and a belt across the hip joint. The axis of the dynamometer was then aligned to the anatomical knee axis, and the lever arm was attached 2–3 cm above the lateral malleolus. Before measuring isometric torques, passive tension of the right knee extensor muscle group was measured at 5, 30, 60, 90, 180, and 270 degrees/sec as an index of spasticity^17^. The test was administered first with SCES OFF and was then repeated after 2–3 min of rest in the same order with SCES ON. Both torque-time integral (TTI; Nm s/s) and the slope of the rise were measured at different angular velocities to reflect the magnitude of the resistance and the hyperexcitability of the extensor motor neuron pool, respectively^7,17^.

#### Participant’s intention to generate isometric torque

Following implantation in P1 and P2, each participant was asked to kick his leg as strong as possible to measure intentional torque of the right knee extensor with SCES OFF. Participants were asked to induce a maximum voluntary contraction with motivation provided over a period of 3–5 s (SCES OFF). The same test was then repeated with SCES ON following ramping the stimulation amplitude to either submotor or motor thresholds. For the submotor (75% of maximum amplitude) and motor thresholds (100% of maximum amplitude), each participant was tested 2 times separated by 2–3 min of rest. Participants were then asked to completely relax and the SCES was tested at a single or two frequencies based on the outcomes of the spinal mapping (SCES ON). Finally, SCES was turned on and the participant was asked to maximally kick with motivation provided over a period of 3–5 s (SCES ON + participant). The test provided insight on the ability to activate the knee extensors, and the force generated with SCES ON served as a proxy measure for the ability to activate the CNS in persons with motor complete SCI.

#### Electrically induced isometric torque

Isometric torques were then measured using surface NMES configured at current amplitude of 100 mA at frequencies of (20, 40 and 80 Hz) and pulse duration 450 µs^29^. The surface electrodes were placed on the knee extensor muscle group in a similar fashion to the RT program. Electrically induced isometric torques were measured to examine the effects of training on the peripheral neuromuscular kinetics. Similar to previous work, we established a force-frequency curve at BL, P1 and P2.

### Statistical analysis

Considering the small sample size, data were presented for each participant in a case series format (Supplementary Table 2). The mean ± SD was then calculated for each outcome variable at different time points (BL, P1, P2). This was then followed by calculating the mean difference between P1 minus BL or P2 minus BL. We primarily relied on standardized effect size considering the small sample size. The mean difference was divided by the pooled standard deviation. Data from the four participants collapsed to examine the effects of EAW + SCES training on the central and peripheral nervous systems outcome variables. Missing data were estimated for NMES-induced isometric torques using a simple linear regression approach between BL and P1 or BL and P2. All data were captured with SCES ON compared to SCES OFF at different time points during the study.

## Results

Four participants enrolled in the current trial and the entire study timeline and design is highlighted in Fig. 1. The physical and spinal cord injury characteristics at the time of enrollment are listed in Table 1. Three participants (0881, 0883 and 0884) completed all aspects of the trial and the three-time point measurements (BL, P1 and P2). One participant (0882) withdrew approximately 3 months following P1 measures because of medical reasons unrelated to the study procedures that resulted in failure to comply with the scheduled visits. However, 0882 data were not excluded and missed data for NMES-isometric torques at P2 measurements were estimated.

Three participants (#0881, 0882 and 0884) were randomized into EAW + ES + RT group and the fourth participant (#0883) was randomized into the EAW + delayed ES + no RT group (Table 1). Participant 0883 was implanted 6 months following BL as a result of randomization into the EAW + delayed ES + no RT group. Additionally, 0883 participant used the walker throughout the study without advancing to Canadian crutches. Table 2 presents the modified Ashworth scores at the time of admission in the REST-trial^21^. For 0881, 0082, 0883 and 0884 participants, the lowest %EAW-support attained was 45%, 55%, 70% and 70%, respectively.

## Effects of percutaneous SCES implantation on the central drive

### SCES OFF versus SCES ON

**Fig. 2:**
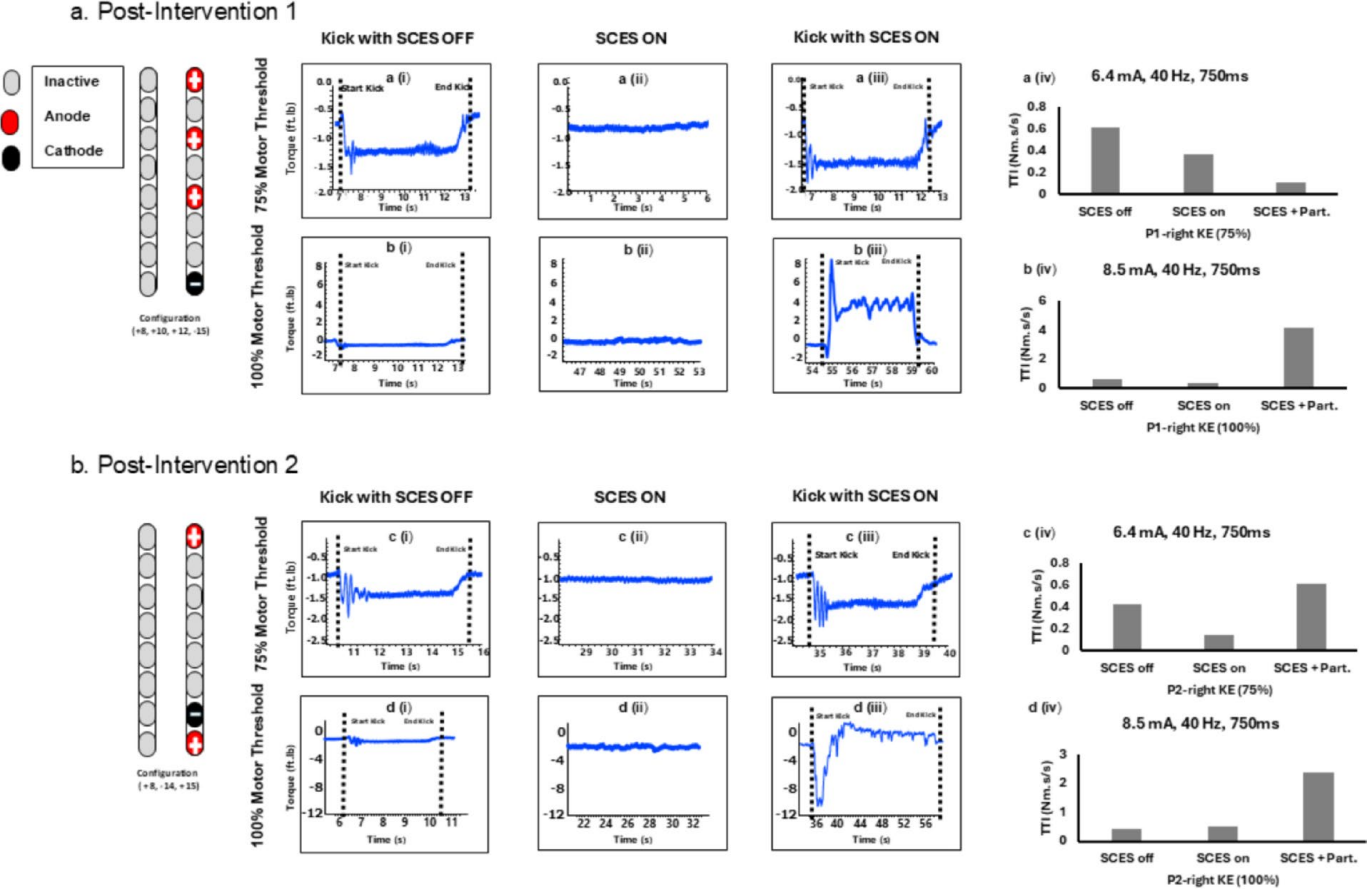
Representative torque-time integral (TTI; Nm s/s) of the right knee extensor (KE) muscle group at three different attempts (kick with SCES OFF, SCES ON and kick with SCES ON) for participant # 0884. The kick with SCES ON (SCES ON + active movement) was performed at 75% submotor threshold or at 100% resting motor threshold during post-intervention (P1) and post-intervention 2 (P2). *Note, Participant 0884 was not implanted at BL*. During kick with SCES OFF, participant was instructed to maximally contract his right KE muscle group over 5 s with motivation provided. During SCES ON, participant was asked to completely relax and the SCES was turned on at the desired configuration to examine the effect on KE torque. Kick with SCES ON, after a resting period of 2–3 min, the SCES was turned on at the desired configurations and then participant was instructed to maximally contract his right KE over a period of 5 s with motivation provided from a research scientist (i.e. active movement). For P1, kick at either 75% (Panels a-i to a-iv) or 100% motor thresholds (Panels b-i to b-iv). Panel a-iv (TTI at 75% submotor threshold during P1) indicated that participant 0884 was able to generate torque in the flexion direction with SCES OFF. Right KE torque decreased to 0.37 Nm s/s (SCES ON) and then subsequently decreased to 0.1 Nm s/s (SCES ON + active movement). Flexion TTI is denoted with negative direction. Panel b-iv (TTI at 100% motor threshold during P1) indicated that participant 0884 generated torque in the flexion direction with SCES OFF. Right KE torque decreased to 0.37 Nm s/s (SCES ON) and then subsequently TTI increased in the extension direction to 4.16 Nm s/s (SCES ON + active movement). Participant 0884 generated 16 × greater extension torque than only SCES ON (3.92/0.24 = 16). Extension TTI is denoted with positive direction. For P2, kick at either 75% (Panels c-i to c-iv) or 100% motor thresholds (Panels d-i to d-iv). Panel c-iv (TTI at 75% submotor threshold during P2) indicated that 0884 participant generated torque in the flexion direction with SCES OFF. Right KE torque decreased to 0.14 Nm s/s (SCES ON) and then subsequently decreased to 0.62 Nm s/s (SCES ON + active movement). Flexion TTI is denoted with negative direction. Panel d-iv (TTI at 100% motor threshold during P2) indicated that participant 0884 generated torque in the flexion direction with SCES OFF. Right KE torque was maintained at 0.51 Nm s/s (SCES ON) and then subsequently TTI increased in the extension direction to 2.39 Nm s/s (SCES ON + active movement). Participant 0884 generated 3.7 × greater extension torque than only SCES ON (1.88/0.51 = 3.7).

The effects of percutaneous SCES on knee extensor isometric torque were tested at 75% (sub-motor threshold) and 100% resting motor threshold stimulation (Fig. 2). Participant 0883 did not generate any response with SCES ON or SCES OFF during his P2. Participant 0883 was not tested at BL because of the delay-entry approach of his group assignment and at P1 due to failure operating the Biodex-dynamometer and the data were not estimated. The amplitude of SCES-submotor and motor thresholds were 3.5 ± 2.6 mA and 4.7 ± 3.4 mA, respectively, remained unchanged for P1 and P2 measurements.

Two participants (0882 and 0884) intentionally (SCES ON + active movement) generated knee extensor isometric torques at either 75% sub-motor threshold (0882; 1.87–6.6 × greater than SCES ON without active movement) or 100% motor threshold [0882 (1.6 × greater than SCES ON without active movement) and 0884 (3.7–16 × greater than SCES ON without active movement)]. Participant # 0884 generated isometric extension torque 16 × and 3.7 × greater than SCES ON without active movement at P1 and P2, respectively (Fig. 2b-iv and d-iv) only at 100% motor threshold. Table 3 presents qualitative interpretation based on the induced isometric torques into flexion and extension directions as a proxy measurement of CNS throughout the study.

**Table 3:** Interpretation of the effects of percutaneous SCES on the central nervous system as measured by the intent to generate isometric peak torque at sub-motor or motor thresholds.

	SCES-75% of motor threshold						SCES-100% of motor threshold					
	BL		P1		P2		BL		P1		P2	
	Cent Flex	Cent Ext	Cent Flex	Cent Ext	Cent Flex	Cent Ext	Cent Flex	Cent Ext	Cent Flex	Cent Ext	Cent Flex	Cent Ext
0881 (20 Hz)	Yes	No	Yes	No	No	Trivial	Yes	No	Yes	No	No	Trivial
0881 (25 Hz)	–	–	Yes	No	Trivial	Trivial	–	–	Yes	No	No	Trivial
0882 (20 Hz)	No	Yes	No	Yes	–	–	Yes	No	Yes	No	–	–
0882 (40 Hz)	No	Yes	Trivial	Trivial	–	–	No	Yes	Yes	No	–	–
0883 (10 Hz)	Not implanted	Not implanted	–	–	No resp.	No resp.	Not implanted	Not implanted	–	–	No resp.	No resp.
0884 (40 Hz)	Not implanted	Not implanted	Yes	No	No	Yes	Not implanted	Not implanted	No	Yes	No	Yes
Summary	1 Yes: 1 No	1 No: 1 Yes	3 Yes: 1 No	3 No: 1 Yes	No Resp.	1 Yes	2 Yes: 1No	1 yes: 2 No	2 Yes:1No	2 No: 1 Yes	No resp.	No resp.
Central drive	Yes		Yes		Yes		Yes		Yes		No	
Pattern	Equal flexionand extension patterns		Flexion pattern		Extension pattern in 1 person		Flexion pattern		Flexion pattern		No resp.	

Cent, Central; Flex, Flexion direction; Ext extension direction; No resp, No response with SCES on; Trivial response, Magnitude of isometric torques was negligible; – indicated that the procedure was not performed, or the Biodex-dynamometer was not functional.

## Effects of percutaneous SCES implantation on peripheral nervous system

### H-reflex: M-wave (H-max/M-max)

**Fig. 3:**
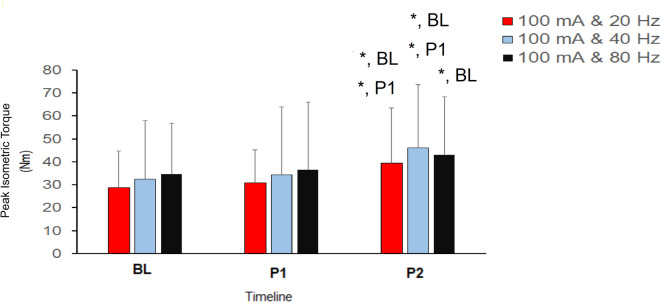
Panel A represents raw H-reflex (mV) and M-waves (mV) motor response (y-axis) that were collected after stimulation with representative current amplitudes (30, 36 and 40 mA) at different timepoints of the study (BL, P1, P2). Panel B represents summary of the soleus muscle H-reflex and M-waves (motor response normalized to M-max; y-axis) in a standing position for the 4 participants in response to the changes in current amplitude (mA; x-axis) at BL, P1 and P2. Participant0882 did not complete because of withdrawal 3 months following P1. A single monophasic pulse was applied at 1000 µs and then current was gradually ramped up from 1–40 mA. All data points were normalized to M-max value at each corresponding timepoint. For 0881, participant’s H -max was achieved at P1 and P2, compared to BL. For participant 0882, H- max attained at P1 similar to BL.

Figure 3 presents H-reflex and M-wave data at BL, P1 and P2 in standing position for the 4 participants. H-reflex data were adjusted to M-max at different time points. Except for 0883 participant, the tests yielded physiological data that reflected changes in the peripheral nervous system in response to training (Fig. 3). The H-max/M-max ratio did not change over time. Individual data indicated improvement in H-reflex and M-waves following P1 (0881 and 0882) and P2 (0881and 0884) (Fig. 3).

### Surface NMES on peak isometric torque

**Fig. 4:**
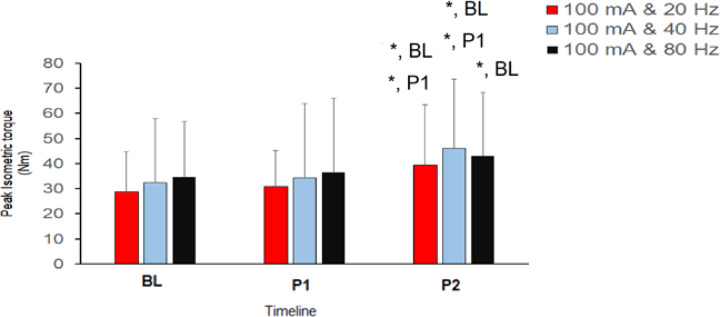
NMES-isometric induced torque (Nm) of the right knee extensor muscle group at 100 mA with different frequencies (20, 40 and 8 Hz). *, P2 statistically different from either BL or P1 (Paired t-tests were used at *P* < 0.05).

Figure 4 presents the results of surface NMES-induced isometric knee extensor peak torques at different frequencies (20, 40 and 80 Hz) at BL, P1 and P2 for the four participants. NMES-peak isometric torques were 37% (*P* = 0.03), 42% (*P* = 0.0005) and 24% (*P* = 0.01) greater at P2 compared to BL at frequencies of 20, 40 and 80 Hz, respectively.

## Effects of percutaneous SCES implantation on exoskeletal performance, EMG-during 10-m walk test and spasticity

### Exoskeletal performance and speed

**Fig. 5:**
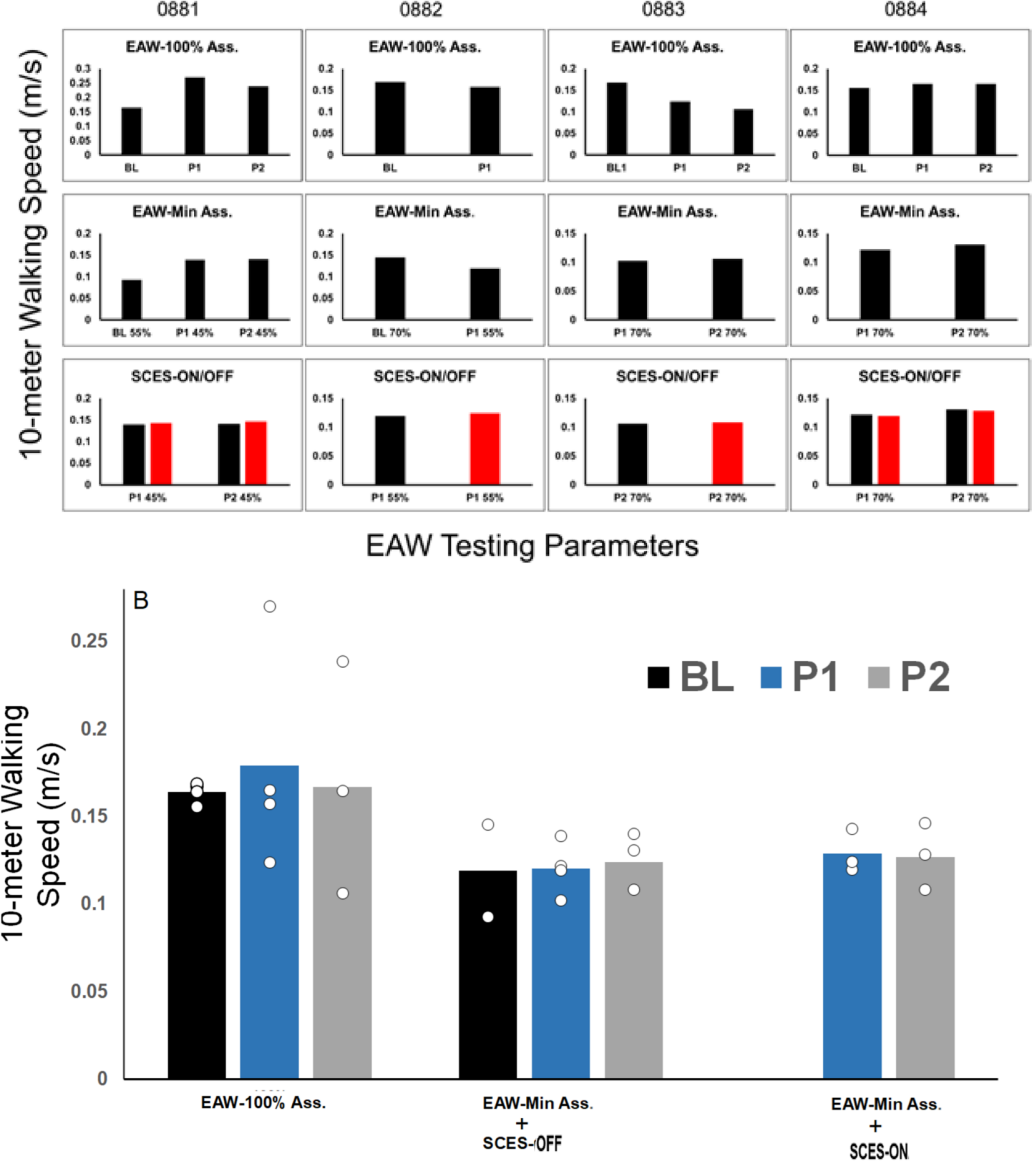
Exoskeletal assisted walking (EAW) performance during 10-m walking test for the four participants at BL, P1 and P2. Panel (**A**) represents 10-m walking speeds (m/s) (y-axis) of each participant at three testing points with different EAW parameters (x-axis) including EAW-100% assistance, EAW-minimum assistance (based on the achievement of each participant), and EAW + SCES ON versus off. Red bars indicate the result with EAW + SCES ON. Panel (**B**) represents the mean of 10-m walking speeds (m/s) of the four participants and the dispersion of dots representing individual data points across different EAW parameters. Mean test results for BL, P1, and P2 are shown by black, blue, and grey bars, respectively. Minimum EAW assistance utilized during testing differs between participants and across testing points, varying from 45 to 70%.

Figure 5 presents the EAW during 10-m walking test as a function of walking speed and time for the four participants. Decreasing EAW assistance (EAW + SCES OFF) decreased walking speed and increased walking time at BL and P2. During P1, EAW + SCES OFF resulted in 37% longer duration (*n* = 4) and 32% slower speed (*n* = 4) compared to 100% EAW + SCES OFF in the 4 participants. Addition of SCES (EAW + SCES ON) did not influence the time and speed compared to EAW + SCES OFF. However, EAW + SCES ON resulted in 30% longer duration (*n* = 3) and a trend of 29% slower speed (*n* = 3) compared to 100% EAW + SCES OFF during P1 measurements (Fig. 5). In P2, the pattern was maintained similar to P1 but was attenuated as far as walking duration (21%; *n* = 3) and speed (24%; *n* = 3).

### Electromyography (EMG) recording during 10-m walk test

**Fig. 6:**
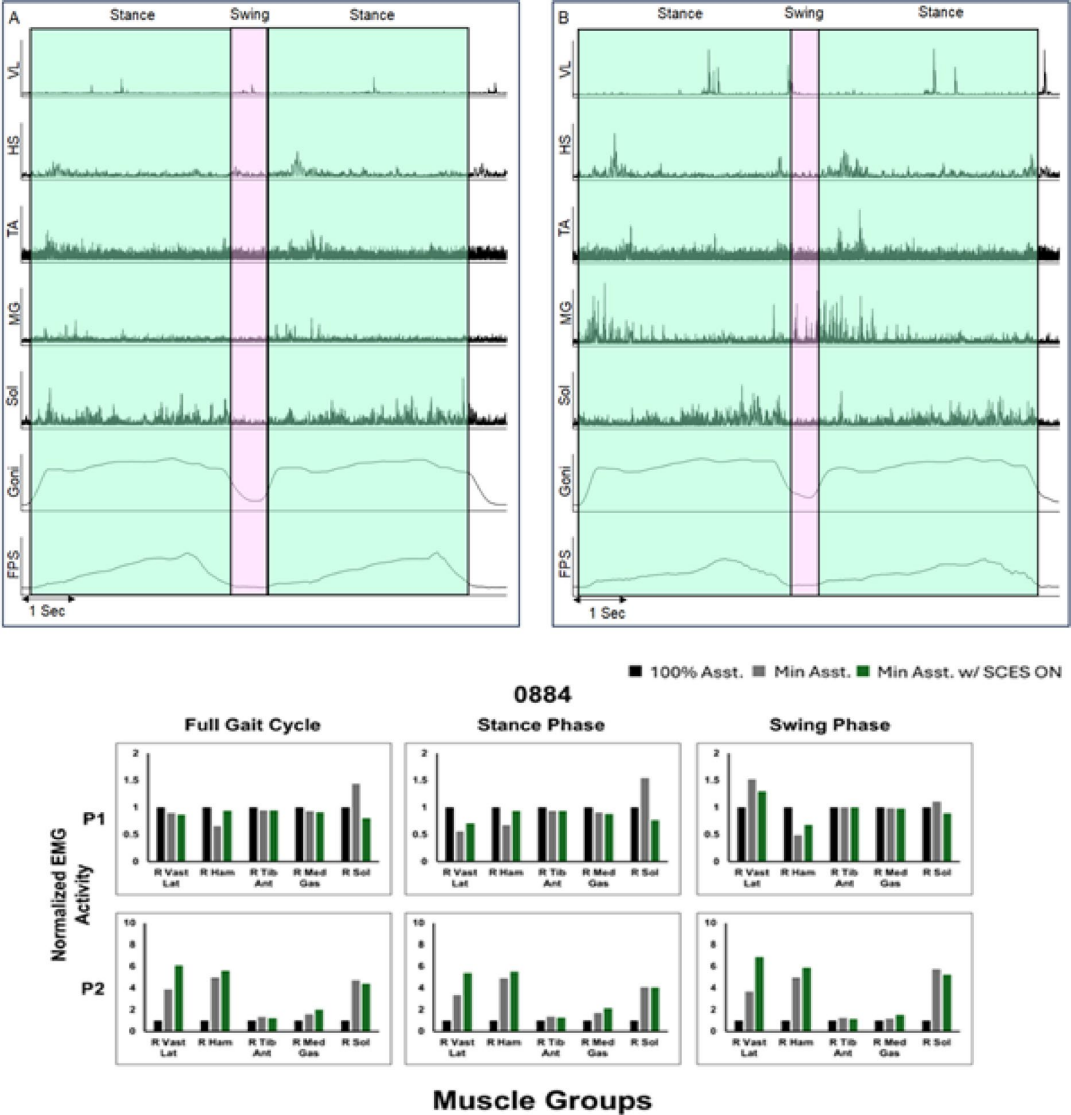
Representative EMG traces from the right leg of participant 0884 during the 10-m Walk Test (10-MWT) during 70% exoskeleton-assisted walking (EAW), collected at testing point P2. Panel (**A**) shows data from the 70% EAW condition with SCES OFF and Panel (**B**) shows data from the 70% EAW condition with SCES ON. Each panel displays two stance phases (highlighted in green) and an intervening swing phase (highlighted in pink). EMG signals were recorded from the following muscles, top to bottom: Vastus Lateralis (VL), Hamstrings (HS), Tibialis Anterior (TA), Medial Gastrocnemius (MG), and Soleus (Sol). Kinematic and temporal reference data include Goniometer (Goni) and Foot Pressure Sensor (FPS). All EMG signals were rectified and acquired using LabChart software. Panel (**C**) demonstrates normalized EMG activities in the full gait cycle, stance phase, and swing phase for participant 0884 during the 10-MWT. Data are presented by muscle groups (right VL: right vastus lateralis, HS: right hamstrings, TA: right tibialis anterior, MG: right medial gastrocnemius, Sol: right soleus). EMG activity is normalized to the EMG activity at 100% EAW for BL, P1, and P2 testing periods. The black bars represent the baseline EMG activity for each testing condition (100%-EAW assistance), grey represents the EMG activity at EAW minimum assistance (70%) with SCES OFF, and green represents EMG activity at EAW-minimum assistance (70%) with SCES ON. *Note* The y-axis was scaled from 0 to 2 in P1 primarily for the purpose of clarity.

Figure 6 summarized the EMG muscle activities during EAW-10-m walking test at different timepoints (BL, P1 and P2). In the stance phase, the EMG amplitude of the vastus lateralis (VL) and tibialis anterior (TA) muscles showed increases accompanied with decreases in hamstrings (HS), medial gastrocnemius (MG) and soleus (SOL) muscles. The EMG amplitude was calculated as the difference between SCES ON and SCES OFF following reduced %EAW assistance during P1 measurements (Fig. 6). The EMG activity increased for VL, HS, MG and SOL muscles and decreased for TA muscle in P2 during the stance phase. VL and MG EMG showed robust increases in P2 compared to P1 during the stance phase. Compared to BL, EMG activities increased in the major muscle groups during P1 and P2. P2 measurements indicated greater EMG activities than P1 during the stance phase.

In the swing phase, the EMG amplitude of VL and MG muscles showed increases, and the EMG of the remaining muscles decreased as a difference between SCES ON and SCES OFF with reduced EAW during P1 measurements (Fig. 6). The EMG activity increased for VL, HS and MG in P2 during the swing phase. The VL and HS muscles showed the robust increase in P2 compared to P1 during stance phase.

### Spasticity performance

**Fig. 7:**
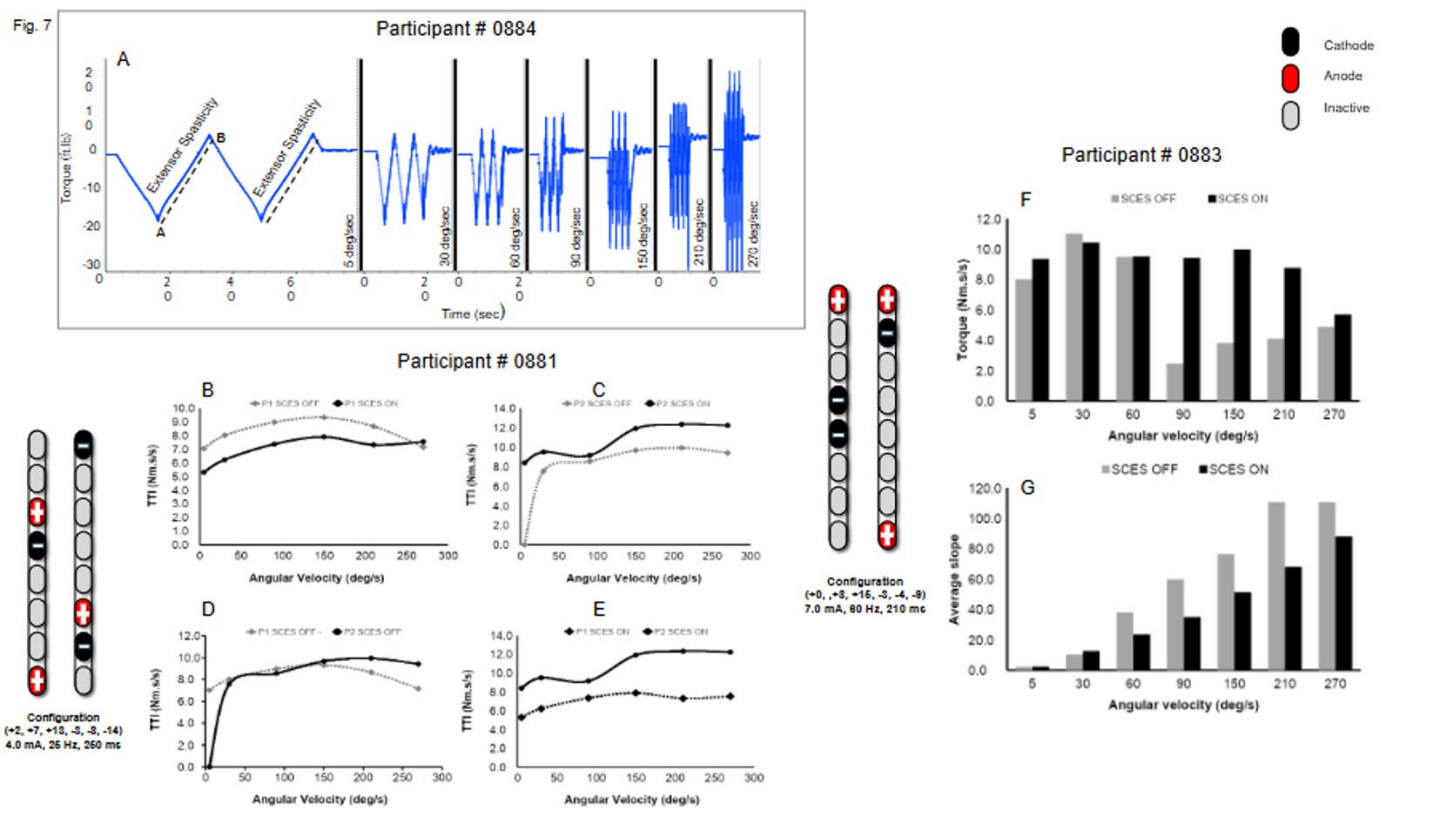
Representative figure demonstrating a step-by-step procedure of analysis of the extensor spasticity in persons with SCI. (**A**) demonstrates A-B dashed line that represents the movement of Biodex arm into flexion direction to measure the passive extension torque (A-B extension torque-resistance to passive movements). Each angular velocity (5–270 deg/sec) was repeated twice and then data were averaged to provide the extensor torques and extensor slopes (data not shown). Torque-time integral (TTI; Nm s/s) was calculated as the area under extensor curve after considering the time elapsed to complete the range of motion from points A to B. (**B**–**E**) represent TTI at 4 different testing conditions with either SCES OFF or SCES ON in participant 0881. Panel (**E**) represents SCES ON at P1 and P2 following the addition of 12 weeks of NMES-RT. Panel (**F**) and (**G**) represent data from participant 0883 at P2. Participant 0883 showed reduction in hyperexcitability with SCES ON (Panel G; decrease in the steepness of the slope at different angular velocities), but not at TTI (Panel F). Note that none of the participants that were admitted to the trial were on anti-spastic medications.

Figure 7 presents the spasticity-angular velocity data starting with 5 deg/sec-270 degree/sec. Spasticity measurements were conducted at P1 (0881 and 0884) and P2 (0881, 0883 and 0884) with SCES OFF and SCES ON, allowing each participant to serve as his own control. Spasticity measurements for 0882 were delayed in P1 before withdrawal from the trial and prior to completing any of the measurements at P1 or P2. 0883 was not tested at BL or P1 because of his allocation to the delayed entry approach group. All spasticity- angular velocities were tested for all participants as previously indicated.

At P1, spasticity-torque time integral (TTI) decreased (0.5%-13%) at different angular velocities. Surprisingly at P2, spasticity-TTI increased (13.5%-41%) at two angular velocities (5 and 270 deg/s). The slope of the rise of the extensor spasticity (5 deg/sec to 270 deg/sec) increased at P1 (13–21%) and P2 (16–44%) during SCES ON compared to SCES OFF.

## Discussion

The major objective of the current pilot work was to examine the effects of SCES implantation in conjunction with EAW training on the adaptations of central and peripheral nervous systems in persons with chronic SCI. Furthermore, we examined whether the effects on the nervous system would translate into motor performance as measured by the 10 m-EAW, changes in EMG between SCES ON and SCES OFF during stance and swing phases of the gait cycle, NMES-induced torque and spasticity as objectively measured in a velocity dependent manner. Training was conducted over the course of 12 months and involved EAW and stand specific training (3 × weekly). Training was then progressed following P1 to include RT in the form of either an open or closed-kinematic chain exercise. Measurements were conducted 6 months apart and included BL, P1 and P2.

The preliminary findings indicated that three participants were capable of generating more extensor torque with SCES ON compared to SCES OFF. However, the fourth participant failed to generate any isometric torque with SCES ON. Participant # 0884 generated isometric peak torque 16 × and 3.7 × greater than only SCES ON without active movement at P1 and P2, respectively. This may reflect the capability of SCES to physiologically magnify the descending supraspinal signals or spinal peripheral signals in persons with motor complete AIS A and B SCI. However, the absence of direct measures that examine supraspinal connectivity, like TMS or fMRI, may limit our hypothesis of the central contribution of the induced isometric torque. Participant 0884 managed to attain standing performance before spinal migration dampened EMG activities of the muscles of the hip joints and subsequently lost independent standing^23^. Previously, Wagner et al. showed similar responses of generating isometric torques at different lower extremity joints in three persons with incomplete AIS C and D^30^. Participants were only capable of generating isometric torque with SCES ON, but not with SCES OFF^30^.

Two important points were noted in the current findings. First, two participants generated isometric peak torques in the flexion direction at baseline. However, this has changed following training, especially at P2, suggesting that training has changed the directionality of generating isometric torque or facilitated reciprocal inhibition of the knee flexors. It is possible to assume that addition of RT following P1 increased the size of the extensor motor neuron pool allowing better activation of the knee extensor torque. Knee extensor muscle may lose up to 50% of its original size in persons with complete SCI^31^.

The second point is that not all participants could generate isometric peak torque with SCES ON similar to 0883. We also failed to elicit a physiological H-reflex measurement in participant 0883 either at BL or P1. The findings may suggest that an intact PNS is essential to drive the supraspinal signals to generate isometric peak torques. The significance of intact proprioceptive feedback circuits has been previously highlighted both in the murine and human models with SCI^8,20^. Additionally, failure to elicit isometric peak torque in 0883 may be related to the extent of migration of the leads that were noted in this participant^23^. We previously used MRI to reconstruct the lumbosacral segments of the spinal cord and noted that we only managed to cover L1-L2 segments with implantation in 0883 participant^23^. Failure to attain target stimulation to L2-L4 segments may explain the lack of knee extensor isometric peak torque following implantation in 0883.

A frequency that induced tonic activation of the extensor muscles was tested at either 75% submotor threshold or 100% resting motor threshold with SCES ON to alter the level of motor unit recruitment^20,32^. It was unclear whether stimulation at 75% of submotor threshold would enable motor unit recruitment in participants with motor complete SCI^20^. On the contrary, 100% motor threshold is likely to induce recruitment of the target motor units. Furthermore, 75% sub-motor threshold may have decreased co-activation of the antagonist motor units. Co-activation may either reciprocally inhibit knee extensor torque or mechanically dilute the magnitude of the resultant torque in the extension direction. However, participant 0884 induced isometric peak torques at 100% motor threshold compared to 75% submotor threshold. The exact mechanism(s) on how motor unit recruitment may influence the induced knee extensor torque is still speculative^20^.

### Effects of the intervention on PNS

Another notable finding is that EAW + SCES induced individualized adaptations on the PNS as measured by H-reflex and M-wave^33^. After adjusting to M-wave max^26–28^, participants 0881 and 0882 showed typical adaptations in response to EAW + SCES ON. The findings suggested that participant 0881 showed improvement in standing H-reflex at P1 and P2 compared to BL measurement. On contrary, we did not elicit a physiological H-reflex in 0883 participant with attenuated H-reflex responses in participant 0884. Participant 0883 has edematous legs as a result of chronic cellulitis that may have interfered with the measurements. On the contrary, participant 0884 did not seem to have any exaggerated spasms or spasticity at the time of admission, which may explain the attenuated H-reflex response in chronic SCI^28^. Additionally, a higher SCES dose (time x energy) may be needed to prime either the CNS or PNS to induce neuroplasticity after SCI. Primarily, the dose was limited to the time of training during the trial. Another plausible explanation is that we did not account for the background EMG activities of the soleus muscle during standing which may provide insights on the differences in H-reflexes responses among the four participants. The rationale is that EMG background activity of the soleus muscle is modest in standing position with maximum support of the exoskeleton. We have also applied a high pass filter with a cutoff frequency 0.3 Hz to set the baseline to 0 mV at BL, P1 and P2 measurements.

Participant 0884 H-max/M-max increased following the addition of RT in the second phase of the study. The rationale of adding RT was previously described and aimed to enhance the PNS circuitry via increasing the size of the motor neuron pool in persons with SCI^21^. Based on previous work, H-reflex and M-wave measurements were conducted in standing position^34–36^. It is well established that the H-reflex is attenuated in standing compared to sitting position^34^. Considering the completeness of the injury, participants used a standard walker to maintain an upright position while using the standing mode of exoskeleton during stimulation of the soleus muscle to elicit H-reflex. It is unclear whether the standing position with a walker may have resulted in unloading the lower extremity muscles to accurately measure soleus H-reflex in participants 0883 and 0884. The findings may also suggest that the H-reflex may serve as a future pre-screening criterion for enrollment of the participants in a similar trial. Absent or attenuated H-reflex may require a period of conditioning to ensure enhancement of the reflex prior to implantation^36^. Those demonstrate improvements in the H-reflex may experience different levels of motor control after SCES implantation.

### Electrically induced torque and PNS

The current preliminary findings suggested that EAW + SCES ON resulted in adaptations of the PNS as determined by NMES-evoked isometric torques at different frequencies^29^. A limitation is that 12 weeks of NMES-RT was introduced in the second phase of the trial. We cannot rule out the effects of EAW + SCES ON from RT on isometric peak torque. However, participant # 0883 showed improvements in isometric peak torques without undergoing 6 months of RT. Similar to previous work^29^, we developed a modified force-frequency curve via applying three different frequencies (20, 40 and 80 Hz). This provided a clear understanding of the calcium-kinetics following training in response to fixed short pulse-duration of 450 µs at a constant amplitude of 100 mA. We recently examined the force-frequency curves at two different amplitudes (50 and 100 mA)^35^. However, our findings indicated that 100 mA is more stable and would result in more homogenous isometric peak toques at different NMES-frequencies compared to 50 mA^38^. Considering the findings, we believe that EAW + SCES ON resulted in neuromuscular adaptations in persons with complete SCI.

### *Implications of using EAW* + *SCES*

Several trials demonstrated the potential benefits of using EAW in enhancing motor recovery in persons with SCI^24,39,40^. These trials demonstrated that EAW could be simply used as a platform with neuromodulation to further enhance neuroplasticity in persons with SCI^7,21,24^. A key aspect is that EAW may result in a reduction of the metabolic cost of walking and allow unlimited steps and repetitions^41^, which are considered important principles in neuroplasticity after SCI^42^. A caveat is that EAW may result in altering gait kinematics that are unlikely to be easily translated in overground ambulation^43,44^. The merging of EAW with neuromodulation modalities has been previously examined in number of case reports^7,17,24,45^. A recent trial demonstrated successful closed-loop application between SCES and EAW in a person with T12 complete SCI^45^. We previously noted that EAW + SCES resulted in an increased ratio of walking time to standing time even with a decreased level of actuator assistance provided by the exoskeleton over the course of 12 weeks^24^. Similarly, our participants required less EAW assistance which may indicate motor improvement in persons with motor complete SCI. In the current trial, decreasing the EAW assistance level subsequently decreased the speed and increased the duration to finish the 10-m walking test. Surprisingly, EAW + SCES ON did not interfere with the gait speed or duration at reduced assistance level. A plausible explanation is that SCES was used at sub-motor threshold to ensure a neuromodulatory effect of the spinal cord-locomotor centers during 10-m EAW test.

The EAW performance was mirrored by increasing EMG activities across different muscle groups in a sequential pattern from BL to P1 and P1 to P2. It is interesting to note the EMG interplay of the lower leg muscle groups (TA, MG and SOL). In P2, the EMG activities of the TA decreased while activity increased in both the MG and the SOL muscles during stance phase. This pattern was reciprocated in the swing phase, where activity increased in the TA, mirrored by a decrease in the EMG activities of the MG and SOL muscles. The findings may suggest that 12 months of EAW + SCES ON improved the interplay of the EMG firing during different phases of the gait cycle. This interplay may suggest dynamic activation of segmental spinal interneurons with EAW + SCES ON^46^.

### Effects of the intervention on spasticity

In the current trial, we investigated the acute (SCES ON compared to SCES OFF) and. the chronic training effects (BL, P1 and P2) on spasticity. The current trial demonstrated that acute effect is likely to be recognized compared to training or chronic effects. The acute effect refers to immediate effect with SCES on, whereas chronic effect refers more to the training effect over time compared to BL (P1 and P2) with SCES OFF. Only with SCES ON, TTI and the slope showed decreases compared to SCES OFF. Previously, SCES managed to decrease flexor and extensor spasticity after using rhythmic configurations^17^. The rhythmic configurations were primarily used to promote EAW performance and overground ambulation in two persons with motor complete SCI^7,17^. Configurations that enhanced task-specific training or standing performance were utilized to determine the effects of the intervention as anti-spastic agent at different timepoints (Fig. 7). Both TTI and slopes were used to examine the effects of SCES on spasticity^47^. TTI indicated the magnitude of resistance at different angular velocities, whereas the slope reflects the level of hyperexcitability of the motor neuron pools of the knee extensor muscle group^47^. Following SCI, spasticity is characterized by hyperexcitability as result of loss of inhibitory descending control^48,49^. It is plausible to assume that the addition of RT program has altered or masked the effects of EAW + SCES training on the hyperexcitability of knee extensor motoneurons. However, a recent work refuted this fact and showed that electrically evoked muscle hypertrophy is unlikely to exacerbate spasticity syndrome in men with chronic SCI^47^. Fortunately, the increase in TTI was only noted at angular velocities of 5 or 270 deg/sec; which is not typically the angular velocities in which human locomotion occurs at speed of 0.4–0.9 m/s^50^. Another potential explanation is that small sample size (*n* = 3) may have inflated the error of measurements at very low and high-angular velocities.

### Limitations and future directions

We have identified several potential limitations that likely influenced the outcomes of the current pilot trial. Limitations include the small size and the inherent heterogeneity that exists in this population. Similar to previous reports^10–12^, the sample size ranged from 1 to 4 participants because of budgetary constraints and the time devoted for rehabilitation in persons with SCI Furthermore, the exclusion criteria were broad, which may reduce generalizability to the entire SCI population. The duration of the trial exceeded 12 months and may likely discourage more participants from committing to the current trial^21^. The rationale of extending the trial to 12 months was primarily based on previous reports after SCI^10–12^. Our current trial is considered a proof-of-concept and is likely to result in designing future studies as short as 6–7 months to decrease participant’s burden; especially with emerging reports that demonstrated standing and stepping within a few days-weeks after implantation^10,30^. Spinal mapping is a lengthy process and may last for 2–3 weeks per participant to decipher the optimum configurations and stimulation parameters for standing and stepping^7,21^. We have currently adopted several scientific approaches to ensure successful completion of this process in future participants^22,23^. This includes MRI construction of spinal lumbosacral segments to ensure accurate placement of the leads and using temporary mapping as a process to guide permanent placement in persons with SCI^23^. Finally, we have developed rigorous strategies to anchor the leads and minimize migration, which may potentially influence the outcomes of the current trial. Several techniques of anchoring percutaneous leads during permanent implantation have been previously proposed including mechanical locking device and suturing the lead to the nearby fascia and ligament^51^.

## Summary/conclusion

Considering the pilot nature (*n* = 4), the current findings noted a disconnect between improvement in the CNS and PNS following EAW + SCES implantation and the translation into motor performance. The EMG patterns during different phases of the EAW gait cycles at P1 and P2 reflect improvements in the CNS performance. Furthermore, the precision to induce SCES extension peak torque has improved over time compared to BL. Three out of the 4 participants noted improvement in the ability to induce volitional exertion with SCES ON. This was mirrored by improvement in the interplay of EMG activities of the lower limb agonist and antagonist muscles during different phases of the gait cycle despite decreased EAW assistance. Participants who intentionally managed to induce isometric torque beyond SCES ON showed different levels of motor improvement as previously documented^7,24^. Participants 0881 and 0882 demonstrated the ability to restore motor control below the level of injury in the form of trunk control and securely restore independent standing as well as stepping, respectively^7^. Participant 0884 demonstrated an ability to restore standing with SCES ON prior to occurrence of migration that impacted his functional standing^24^.

Intact PNS is highly important to induce central adaptation to be translated into motor control. Absence of PNS adaptations in participant 0883, as demonstrated by the lack of measurable H-reflex to M-max ratio, was accompanied by lack of CNS adaptations or enhanced motor behavior. Furthermore, the improvement in PNS (0881) over time was accompanied by decreasing spasticity at different angular velocities. However, participant 0883 demonstrated decreases in the hyperexcitability component of spasticity with EAW + SCES ON. The findings may suggest possible release of inhibitory neurotransmitters without altering PNS in persons with SCI^49^. At the time of the admission, only 0881 and 0883 participants demonstrated noticeable modified Ashworth scores without receiving anti-spastic medications. Both participants demonstrated an acute effect with SCES on the magnitude and hyperexcitability levels of spasticity, respectively. In general, the effects of SCES on spasticity are still controversial and may warrant additional investigations.

The improvements in NMES-induced isometric torques may reflect changes in the neuromuscular kinetics at P2, regardless of the group assignments. This may suggest adaptations in the neuromuscular component of the PNS after SCI. Overall, the disconnect in the current findings may reflect the specificity of the spinal cord to the SCES configurations or to the applied training paradigm. Finally, the addition of SCES to EAW did not affect the 10-m walking performance. SCES was applied at sub-motor thresholds during EAW to intentionally induce steps with reduced assistance. The current findings managed to partition the adaptations in the primary components of the nervous system in relation to motor performance after EAW training and SCES implantation. However, we should exercise caution in assuming causality considering the small sample size and the need for large clinical trials.

## Supplementary Information

Below is the link to the electronic supplementary material.

Supplementary Material 1

Supplementary Material 2

## Data Availability

Data will be shared by the lead contact author [Ashraf Gorgey (ashraf.gorgey@va.gov)] upon request and after obtaining necessary approval from local research office. Version and URL of software used in the study: LabChart 8.1.21 (Windows, A.D. Instruments, Sydney, Australia) (https://www.adinstruments.com).

## Abbreviations

AIS: ASIA impairment scale
CNS: Central nervous system
EAW: Exoskeletal assisted walking
10-MWT: 10-M walking test
NMES: Neuromuscular electrical stimulation
NMES-RT: NMES-resistance training
PNS: Peripheral nervous system
RT: Resistance training
SCES: Spinal cord epidural stimulation
SCI: Spinal cord injury
TTI: Torque time integral
