# Supplementary material for: Partitioning of the nervous system following exoskeleton and epidural stimulation in spinal cord injury
Source: Sci Rep. 2026 May 12;16:21708. doi: 10.1038/s41598-026-52650-0 (PMC13357727; doi:10.1038/s41598-026-52650-0)
Supplement: Supplementary file 1 — Supplementary Material 1 [file 41598_2026_52650_MOESM1_ESM.docx]

Supp Table 1. List of SCES configurations and stimulation parameters that were used throughout the study.

|  | EAW- Training  Phase 1 | EAW-Training  Phase 2 | Torque  SCES-ON | Spasticity  SCES-ON | 10-meter walk test  SCES-ON |
| --- | --- | --- | --- | --- | --- |
| 0881 | 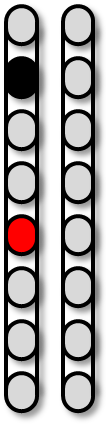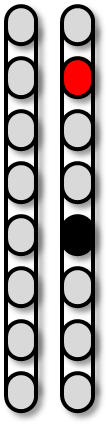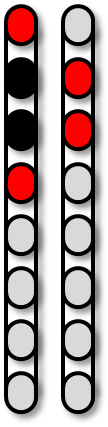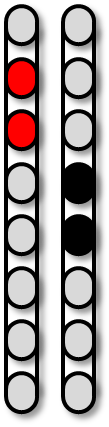 | 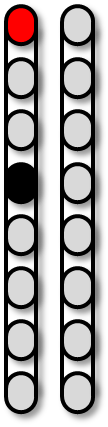 |  |  |  |
| 0881-stimulation Parameters | 1.7-3.5 mA, 350-450 µs, 25 Hz | 3.0 mA, 250 µs, 25 Hz | 2.6 mA. 20 Hz  3.5mA, 20 Hz  2.6 mA, 25 Hz  3.5 mA, 25 Hz | 4.0 mA, 25 Hz, 250 µs | 3.0 mA, 250 µs, 25 Hz |
| 0882 | 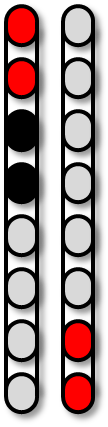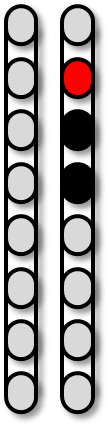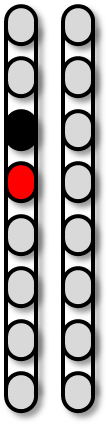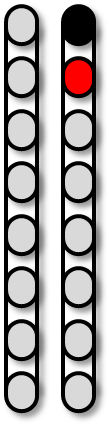 | 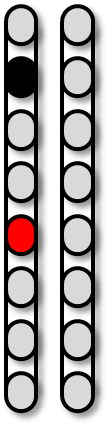 |  | Participant did not undergo spasticity testing |  |
| 0882-stimulation Parameters | 1.6-2.3 mA, 350-450 µs, 20 Hz | 3.5 mA, 250 µs,30 Hz | 1.5 mA, 20 Hz  2 mA, 20 Hz  1.5 mA, 40 HZ,  2 mA, 40 Hz |  | 1.6 mA, 20 Hz,  2.3 mA 20 Hz |
| 0883 |  |  |  |  |  |
| 0883-stimulation Parameters |  | 7 mA, 60 Hz, 300 µs | 6.8 mA, 10 Hz, 370 µs  9.0 mA, 10 Hz | 7.0 mA, 60 Hz, 210 µs | 7 mA, 60 Hz, 300 µs |
| 0884 |  |  | P1 |  |  |
| 0884-stimulation Parameters | 6-8 mA, 210 µs | 6-8 mA, 210 µs | 6.4mA, 40 Hz  8.5 mA, 40 Hz | 7.9 mA, 10 Hz, 750 µs | 6-8 mA, 210 µs |
