# Supplementary material for: Partitioning of the nervous system following exoskeleton and epidural stimulation in spinal cord injury
Source: Sci Rep. 2026 May 12;16:21708. doi: 10.1038/s41598-026-52650-0 (PMC13357727; doi:10.1038/s41598-026-52650-0)
Supplement: Supplementary file 2 — Supplementary Material 2 [file 41598_2026_52650_MOESM2_ESM.docx]

| **Supplemental Table 2a.** Effects of EAW+percutaneous SCES implantation on the central drive as measured by subject’s intention to generate knee extensor isometric torques | | | | | | | | | | | | |
| --- | --- | --- | --- | --- | --- | --- | --- | --- | --- | --- | --- | --- |
| **Torque-Time Integral (TTI; Nm.s/s)** | | | | | | | | | | | | |
|  | **0881** | | | **0882** | | | **0883** | | | **0884** | | |
|  | **20 Hz, 2.6mA (75% MT*)** | | | **20 Hz, 1.5mA (75% MT)** | | |  |  |  | **40 Hz, 6.4 mA (75% MT)** | | |
|  | **SCES off** | **SCES on** | **SCES + 0881** | **SCES off** | **SCES on** | **SCES + 0882** |  |  |  | **SCES off** | **SCES on** | **SCES + 0884** |
| **BL** |  | 2.49 | 0.45 | 1.12 | 0.86 | 0.99 |  |  |  |  |  |  |
| **P1** | 0.02 | 1.64 | 0.06 | 0.12 | 0.74 | 1.16 |  |  |  | 0.61 | 0.37 | 0.1 |
| **P2** | 0.06 | 0.03 | 0.05 | Withdrawn Prior to P2 | | |  |  |  | 0.43 | 0.14 | 0.62 |
|  | **20 Hz, 3.5mA (100% MT)** | | | **20 Hz, 2 mA (100% MT)** | | |  |  |  | **40 Hz, 8.5mA (100% MT)** | | |
|  | **SCES off** | **SCES on** | **SCES + 0881** | **SCES off** | **SCES on** | **SCES + 0882** |  |  |  | **SCES off** | **SCES on** | **SCES + 0884** |
| **BL** |  | 2.73 | 0.13 | 1.12 | 2.55 | 0.45 |  |  |  |  |  |  |
| **P1** | 0.02 | 0.95 | 0.08 | 0.12 | 2.72 | 1.15 |  |  |  | 0.61 | 0.37 | 4.16 |
| **P2** | 0.06 | 0.04 | 0.085 | Withdrawn Prior to P2 | | |  |  |  | 0.43 | 0.51 | 2.39 |
|  | **25 Hz, 2.6mA (75% MT)** | | | **40 Hz, 1.5 mA (75% MT)** | | |  |  |  |  |  |  |
|  | **SCES off** | **SCES on** | **SCES + 0881** | **SCES off** | **SCES on** | **SCES + 0882** |  |  |  |  |  |  |
| **BL** |  |  |  | 1.12 | 1.25 | 0.86 |  |  |  |  |  |  |
| **P1** | 0.02 | 0.36 | 0.12 | 0.12 | 0.12 | 0.1 |  |  |  |  |  |  |
| **P2** | 0.06 | 0.06 | 0.03 | Withdrawn Prior to P2 | | |  |  |  |  |  |  |
|  | **25 Hz, 3.5mA (100% MT)** | | | **40 Hz, 2 mA (100% MT)** | | |  |  |  |  |  |  |
|  | **SCES off** | **SCES on** | **SCES +0 881** | **SCES off** | **SCES on** | **SCES + 0882** |  |  |  |  |  |  |
| **BL** |  |  |  | 1.12 | 1.61 | 0.78 |  |  |  |  |  |  |
| **P1** | 0.02 | 0.54 | 0.04 | 0.12 | 0.48 | 0.19 |  |  |  |  |  |  |
| **P2** | 0.06 | 0.0097 | 0.0052 |  |  |  |  |  |  |  |  |  |
| ***MT: Motor Threshold** | | |  |  |  |  |  |  |  |  |  |  |

**Supplemental Table 2b.** Effects of EAW+Percutaneous SCES implantation on peripheral nervous system as measured by H-reflex, M-wave and H-max to M-max ratio

|  |  |  |  |  |  |  |  |  |  |
| --- | --- | --- | --- | --- | --- | --- | --- | --- | --- |
| **0881** | | | | |  | **0882** | | | |
| **Condition** | | **H-max** | **M-max** | **H-max/M-max ratio** |  | **Condition** | **H-max** | **M-max** | **H-max/M-max ratio** |
| Baseline | | 0.000153 | 6.49E-05 | 2.3575 |  | Baseline | 0.0013 | 0.0019 | 0.7219 |
| P1 (6 months) | | 0.0347 | 0.0166 | 2.0904 |  | P1 (6 months) | 0.2737 | 0.2350 | 1.1648 |
| P2 (12 months) | | 0.7312 | 0.1124 | 6.5053 |  | P2 (12 months) | Withdrawn Prior to P2 | | |

| **0883** | | | |  | **0884** | | | |
| --- | --- | --- | --- | --- | --- | --- | --- | --- |
| **Condition** | **H-max** | **M-max** | **H-max/M-max ratio** |  | **Condition** | **H-max** | **M-max** | **H-max/M-max ratio** |
| Baseline | 0.03 | 0.02 | 1.23 |  | Baseline | 0.47 | 0.88 | 0.54 |
| P1 (6 months) | 0.01 | 0.01 | 1.14 |  | P1 (6 months) | 0.17 | 1.05 | 0.16 |
| P2 (12 months) | 0.01 | 0.01 | 2.19 |  | P2 (12 months) | 0.10 | 0.65 | 0.15 |

**Supplemental table 2c.** Effects of EAW+percutaneous SCES implantation on peripheral neuromuscular kinetics. Surface neuromuscular electrical stimulation was used to stimulate right the knee extensor muscle group at different frequencies [20, 40 and 80 Hz] to induce isometric peak torques (Nm).

|  |  |  |  |  |  |  |  |  |  |  |  |
| --- | --- | --- | --- | --- | --- | --- | --- | --- | --- | --- | --- |
|  | 100mA, 20Hz |  |  |  | 100mA, 40 Hz |  |  |  | 100mA, 80 Hz |  |  |
|  | BL Right | P1 Right | P2 Right |  | BL Right | P1 Right | P2 Right |  | BL Right | P1 Right | P2 Right |
| **0881** | 27.04 | 23.25 | 35.17 |  | 29.95 | 19.32 | 44.09 |  | 29.04 | 23.04 | 39.78 |
| **0882** | 56.19 | 60.89 | 77.05 |  | 66.34 | 71.92 | 82.78 |  | 65.07 | 75.45 | 77.53 |
| **0883** | 9.32 | 10.24 | 12.03 |  | 4.77 | 4.11 | 16.18 |  | 11.77 | 6.74 | 16.64 |
| **0884** | 22.57 | 29.41 | 33.27 |  | 28.62 | 42.12 | 41.22 |  | 32.56 | 40.65 | 38.16 |
| **Mean** | **28.78** | **30.95** | **39.38** |  | **32.42** | **34.37** | **46.07** |  | **34.61** | **36.47** | **43.03** |
| **SD** | **19.76** | **21.50** | **27.22** |  | **25.40** | **29.51** | **27.50** |  | **22.25** | **29.45** | **25.30** |

Highlighted grey values were predicted based on simple linear regression calculations between either BL and P1 measurements or BL and P2 measurements.

**Supplemental table 2d.** Effects of EAW+percutaneous SCES implantation on exoskeletal performance during 10-meter walking test at BL, P1 and P2 in persons with SCI.

| **Timepoints** | **0881** | **Time(s)** | **Speed (m/s)** |
| --- | --- | --- | --- |
| **BL** | 881-BL-100% EAW-No SCES | 60.86 | 0.16 |
|  | 881-BL-55% EAW-No SCES | 107.50 | 0.09 |
| **P1** | 881-P1-100% EAW-No SCES | 37.24 | 0.27 |
|  | 881-P1-45% EAW-No SCES | 71.97 | 0.14 |
|  | 881-P1-45% EAW-With SCES | 69.82 | 0.14 |
| **P2** | 881-P2-100% EAW-No SCES | 41.91 | 0.24 |
|  | 881-P2-45% EAW-No SCES | 71.35 | 0.14 |
|  | 881-P2-45% EAW-With SCES | 68.27 | 0.15 |

| **Timepoints** | **0882** | **Time(s)** | **Speed(m/s)** |  |
| --- | --- | --- | --- | --- |
| **BL** | BL 100% No SCES Stance cycle | 59.125 | 0.17 |  |
|  | BL 70% No SCES Stance cycle | 68.79 | 0.15 |  |
| **P1** | P1 100% NO SCES | 63.455 | 0.16 |  |
|  | P1 55% NO SCES | 80.345 | 0.12 |  |
|  | P1 55% with SCES | 83.68 | 0.12 |  |

| **Timepoints** | **0883** | **Time (s)** | **Speed (m/s)** |
| --- | --- | --- | --- |
| BL | BL 100% No SCES | 59.67 | 0.17 |
| P1 | P1 100% No SCES | 80.75 | 0.12 |
|  | P1 70% No sces | 97.72 | 0.10 |
| P2 | P2 100% No SCES | 94.19 | 0.11 |
|  | P2 70% No SCES | 94.5 | 0.11 |
|  | P2 70% SCES-ON | 92.33 | 0.11 |

| **Timepoints** | **0884** | **Time(s)** | **Speed (m/s)** |
| --- | --- | --- | --- |
| **BL** | BL 100% No SCES | 64.1 | 0.16 |
| **P1** | P1 100% No SCES | 60.605 | 0.17 |
|  | P1 70% No SCES | 82.205 | 0.12 |
|  | P1 70% with SCES ON | 83.47 | 0.12 |
| **P2** | P2 100% No SCES | 60.66 | 0.16 |
|  | P2 70% No SCES | 76.5 | 0.13 |
|  | P2 70% with SCES ON | 78 | 0.13 |

**Supplemental table 2e.** Effects of EAW+percutaneous SCES implantation on spasticity measurements [torque time integral and hyperexcitability as measured by the slope] at BL, P1 and P2 in persons with SCI

| **Torque-time integral (TTI, Nm.s/s)** | | | | | | | | | |
| --- | --- | --- | --- | --- | --- | --- | --- | --- | --- |
| **Participant** | **Testing Point** | **SCES ON/Off** | **5 deg/sec** | **30 deg/sec** | **60 deg/sec** | **90 deg/sec** | **150 deg/sec** | **210deg/sec** | **270 deg/sec** |
| **0881** | P1 | SCES Off | 7.04 | 8.01 |  | 8.98 | 9.32 | 8.67 | 7.16 |
|  | P1 | SCES ON | 5.30 | 6.23 |  | 7.36 | 7.90 | 7.32 | 7.54 |
|  | P2 | SCES Off |  | 7.59 |  | 8.55 | 9.67 | 9.94 | 9.42 |
|  | P2 | SCES ON | 8.40 | 9.51 |  | 9.16 | 11.92 | 12.34 | 12.24 |
|  |  |  |  |  |  |  |  |  |  |
| **0883** |  |  | **5 deg/sec** | **30 deg/sec** | **60 deg/sec** | **90 deg/sec** | **150 deg/sec** | **210 deg/sec** | **270 deg/sec** |
|  | P2 | SCES Off | 8.03 | 9.71 | 6.47 | 2.16 | 1.45 | 2.82 | 4.81 |
|  |  | SCES ON | 9.89 | 10.36 | 9.75 | 9.20 | 9.56 | 8.34 | 7.54 |
|  |  |  |  |  |  |  |  |  |  |
| **0884** |  |  | **5 deg/sec** | **30 deg/sec** | **60 deg/sec** | **90 deg/sec** | **150 deg/sec** | **210 deg/sec** | **270 deg/sec** |
|  | BL | SCES Off | 7.68 | 6.78 |  | 10.75 | 10.96 | 9.52 | 10.71 |
|  | P1 | SCES Off | 9.74 | 11.31 | 13.04 | 12.64 | 12.92 | 11.65 | 12.26 |
|  | P1 | SCES ON | 9.53 | 10.60 | 12.37 | 11.94 | 12.35 | 11.53 | 11.76 |
|  | P2 | SCES Off | 8.53 | 9.91 | 12.60 | 11.54 | 12.94 | 12.01 | 11.87 |
|  | P2 | SCES ON | 10.46 | 11.01 | 13.06 | 12.58 | 14.07 | 12.73 | 13.41 |

| **Average Slope of Torque (Nm/s)** | | | | | | | | | |
| --- | --- | --- | --- | --- | --- | --- | --- | --- | --- |
| **Participant** | **Testing Point** | **SCES ON/Off** | **5 deg/sec** | **30 deg/sec** | **60 deg/sec** | **90 deg/sec** | **150 deg/sec** | **210 deg/sec** | **270 deg/sec** |
| **0881** | P1 | SCES Off | 1.26 | 7.11 |  | 17.69 | 23.29 | 29.38 | 37.09 |
|  | P1 | SCES ON | 1.34 | 7.58 |  | 19.11 | 25.34 | 32.62 | 38.52 |
|  | P2 | SCES Off |  | 1.35 |  | 7.71 | 19.61 | 26.15 | 33.62 |
|  | P2 | SCES ON | 1.60 | 9.93 |  | 9.82 | 27.01 | 36.20 | 45.09 |
|  |  |  |  |  |  |  |  |  |  |
|  |  |  | **5 deg/sec** | **30 deg/sec** | **60 deg/sec** | **90 deg/sec** | **150 deg/sec** | **210 deg/sec** | **270 deg/sec** |
| **0883** | P2 | SCES Off | 1.65 | 14.40 | 27.56 | 31.52 | 58.38 | 79.13 | 93.76 |
|  |  | SCES ON | 1.72 | 9.36 | 17.34 | 27.77 | 40.65 | 53.53 | 71.26 |
|  |  |  |  |  |  |  |  |  |  |
|  |  |  | **5 deg/sec** | **30 deg/sec** | **60 deg/sec** | **90 deg/sec** | **150 deg/sec** | **210deg/sec** | **270 deg/sec** |
| **0884** | BL | SCES Off | 1.9 | 10.5 |  | 26.6 | 34.2 | 42.0 | 51.8 |
|  | P1 | SCES Off | 1.8 | 10.3 | 18.7 | 25.6 | 32.4 | 41.9 | 51.7 |
|  | P1 | SCES ON | 1.7 | 9.9 | 17.9 | 24.5 | 31.3 | 40.5 | 51.7 |
|  | P2 | SCES Off | 1.86 | 10.96 | 21.68 | 27.46 | 35.71 | 45.24 | 56.79 |
|  | P2 | SCES ON | 1.81 | 10.67 | 19.73 | 27.34 | 35.53 | 47.06 | 53.98 |

Highlighted grey reflects measurements that were not captured during for 0881 participant or because in error of recording data during the actual test
